# Effects of antibiotic treatments on symbiotic bacteria and life history traits of *Bemisia tabaci* and *Trialeurodes vaporariorum* (Hemiptera: Aleyrodidae): implications for pest control strategies

**DOI:** 10.1093/jee/toaf136

**Published:** 2025-09-11

**Authors:** Marzieh Kashkouli, Jahangir Khajehali

**Affiliations:** Research Institute for Biotechnology and Bioengineering, Isfahan University of Technology, Isfahan, Iran; Department of Plant Protection, College of Agriculture, Isfahan University of Technology, Isfahan, Iran

**Keywords:** whitefly, antibiotic-mediated disruption, antibiotic cocktail, age–stage 2-sex life table

## Abstract

*Bemisia tabaci* Gennadius and *Trialeurodes vaporariorum* Westwood are damaging agricultural pests, with control complicated by their high reproduction and resistance to treatments. This study investigates the effects of antibiotic treatments on the symbiotic bacteria and life history traits of these whitefly species using primer-specific PCRs, quantitative PCRs (qPCRs), and life table analyses. Results revealed that *B. tabaci* (isolate IUT1) hosts a diverse set of symbionts, including *Portiera*, *Hamiltonella*, *Arsenophonus*, and *Rickettsia*, while *T. vaporariorum* (isolate IUT2) carries only *Portiera* and *Arsenophonus*. The antibiotic treatments—tetracycline, rifampicin, and a cocktail of ampicillin, cefotaxime, and gentamicin—significantly altered the abundance of specific symbionts, with notable reductions of *Rickettsia* in *B. tabaci* and *Arsenophonus* in *T. vaporariorum*. In addition, ampicillin, cefotaxime, gentamicin, and tetracycline treatments resulted in significant decreases and increases of *Portiera* in both whiteflies, respectively. Antibiotic exposure also led to profound effects on whitefly preadult durations, fecundity, life expectancy, and some biological parameters including *r*, *R*_0_, and gross reproductive rate of whiteflies. Specifically, *B. tabaci* treated with rifampicin lost its ability to mature into adulthood, while *T. vaporariorum* treated with rifampicin was unable to lay eggs. Furthermore, treatment with ampicillin, cefotaxime, and gentamicin rendered both species of whiteflies incapable of egg-laying. A negative *r* value was also recorded in *B. tabaci* following tetracycline treatment. These results highlight the critical role of symbiotic bacteria in whitefly biology and provide insight into the potential consequences of disrupting these associations.

## Introduction

*Bemisia tabaci and Trialeurodes vaporariorum* are among the most significant agricultural pests, primarily causing damage through direct feeding and serving as vectors for various plant viruses. These pests are responsible for considerable reductions in crop yields and negatively affect the quality of agricultural products (Johnson et al. 1992, Padilha et al. 2021). The challenges associated with their management underscore the necessity for innovative integrated pest management (IPM) strategies. Several promising approaches based on manipulating symbiotic relationships have been identified. Among them, the disruption of microbial symbionts holds particular potential, as it aims to suppress insect pests by eliminating symbiotic microorganisms required for their growth, reproduction, and survival, and/or by disrupting the vertical transmission of these symbionts to subsequent generations (Baumann 2005, Salem et al. 2015, Kashkouli et al. 2021b). A comprehensive understanding of symbiont diversity and function is crucial for advancing knowledge of insect ecology and for the development of novel pest management strategies.

Whiteflies are associated with a variety of primary (P-symbionts, which are obligatory) and secondary (S-symbionts, which are generally facultative) symbionts that play crucial roles in their survival and fitness (Kashkouli et al. 2021a, 2021b, Kareem et al. 2023, Kashkouli and Khajehali 2024). The primary symbiont identified is *Portiera aleyrodidarum*, which is essential for the nutritional requirements (Baumann 2005, Kashkouli and Khajehali 2024). This symbiont has been identified in various whitefly populations across diverse regions, including Iraq, Iran, the United Kingdom, and Pakistan, underscoring its widespread occurrence and potential ecological significance (Bibi et al. 2016, Kareem et al. 2023, Kashkouli and Khajehali 2024). In addition, several secondary symbionts have been documented, including *Rickettsia* (*Rickettsiales*), *Hamiltonella* (*Enterobacteriales*), *Wolbachia* (*Rickettsiales*), *Arsenophonus* (*Enterobacteriales*), *Fritschea* (*Chlamydiales*), and *Cardinium* (*Bacteroidetes*) (Weeks et al. 2003, Everett et al. 2005, Fujiwara et al. 2023, Kashkouli and Khajehali 2024). Research has demonstrated that these symbionts significantly influence key host characteristics, including insect–plant interactions, host fitness, thermal tolerance, and resistance to pesticides (Shan et al. 2016, Zhao et al. 2018, Liu et al. 2020, Barman et al. 2022, Tan et al. 2023).

Investigating the roles of insect endosymbionts is challenging due to the technical difficulties associated with culturing these microorganisms in vitro. Their highly specialized and fastidious nature, as well as their coevolutionary adaptation to insect hosts, restricts their ability to thrive in artificial environments (Masson and Lemaitre 2020). Consequently, researchers often employ antibiotics or antibacterial reagents to selectively eliminate specific symbionts, thereby facilitating the study of their functions in host physiology (Raina et al. 2015, Tan et al. 2023). The use of antibiotics in whiteflies has been explored as a means of manipulating their endosymbiotic communities. Key antibiotics, including rifampicin, tetracycline, and ampicillin, have been identified, each exhibiting varying degrees of effectiveness in targeting whitefly endosymbionts (Ruan et al. 2006, Ahmed et al. 2010, Xue et al. 2012, Raina et al. 2015, Lv et al. 2018, Milenovic et al. 2022, Tan et al. 2023).

While substantial research has been conducted on the role of symbiotic bacteria in the growth and development of *B. tabaci*, comparatively little attention has been given to the symbionts of *T. vaporariorum* and their functional significance (Kareem et al. 2023). This study aims to investigate the effects of antibiotic treatments on the life table parameters and symbiont populations in both *B. tabaci* and *T. vaporariorum*. The findings will contribute to a more comprehensive understanding of the biological roles of endosymbionts and provide insights into potential pest management strategies for these agriculturally significant species.

## Materials and Methods

### Insect Collection and Morphological Identification of Whiteflies

Populations of *B. tabaci* (isolate IUT1) and *T. vaporariorum* (isolate IUT2) were collected in May 2023 from cucumber greenhouse in Isfahan University of Technology (IUT). The whitefly specimens were morphologically examined for species identification using identification keys (Hill 1969, Martin 1987, European and Mediterranean Plant Protection Organization 2004, Patel et al. 2022, Kashkouli and Khajehali 2024). During this process, the populations were maintained on cucumber seedlings in insect-proof cages within the greenhouse under controlled conditions (25 ± 5 °C and 60 ± 10% relative humidity).

### Insect Rearing

Following collection and morphological confirmation of species identity, the adult whiteflies were transferred to insect-proof cages (90 × 90 × 90 cm) containing cucumber seedlings. These cages were kept in climatic chambers with controlled conditions (25 ± 2 °C, a photoperiod of 14:10 h (L:D), and 60 ± 5% relative humidity) to facilitate colony establishment. The colonies were reared for more than two generations before conducting further research.

### DNA Extraction and PCR Amplification

For DNA extraction, adult whiteflies were surface sterilized by immersing them in 75% ethanol for 2 min, followed by 3 washes with distilled sterile water. Genomic DNA (gDNA) was extracted from the adults using the CTAB method (Murray and Thompson 1980) and stored at −20 °C. The DNA concentration and integrity were assessed by agarose gel electrophoresis (90 V for 90 min), and the gel images were captured using a gel documentation system (Vilber, France). The extracted DNA was then used for subsequent PCR and real-time PCR analyses.

PCR techniques were used to identify whitefly species, *B. tabaci* biotypes, and the genera of obligate and facultative bacterial symbionts associated with the whiteflies. Whitefly species were identified through mitochondrial cytochrome c oxidase I (*COI*) gene markers (Supplementary Table 1). To distinguish between the biotypes B and Q of *B. tabaci*, a microsatellite marker, Bem23-(GAA)31imp (De Barro et al. 2003, Skaljac et al. 2010), was employed in a standard PCR assay, which differentiates the biotypes based on the size of the PCR product fragments (Supplementary Table 1). Furthermore, *16S rRNA* and *23S rRNA* gene markers were used to amplify the bacterial endosymbionts associated with the whiteflies, including *Portiera*, *Arsenophonus, Wolbachia*, *Hamiltonella, Rickettsia*, *Cardinium*, and *Fritschea* (Supplementary Table 1). PCR reactions were carried out in a 25 µl reaction volume, containing the DNA template, specific primers, and PCR master mix. The amplification conditions included an initial denaturation at 94 °C for 3 min, followed by 30 cycles of denaturation at 94 °C for 1 min, annealing at 55 °C for 45 s, and extension at 72 °C for 1 min, with a final extension at 72 °C for 10 min. The sizes of the PCR products were confirmed through agarose gel electrophoresis and visualized with gel documentation. The PCR products were then purified using a Gel Extraction Kit (Favorgen, Yekta Tajhiz Azma Co., Iran) before subjecting for Sanger sequencing.

### Antibiotic Treatments

The antibiotic treatments were administered using an artificial diet-feeding method, employing Parafilm membrane sachets to facilitate direct feeding by adult whiteflies (Bedford et al. 1994, Noda et al. 2001, Ruan et al. 2006). Briefly, a plastic bottle (40 mm in diameter × 85 mm in length), featuring an 8 mm-diameter hole for ventilation, was sealed with a Parafilm membrane stretched as thinly as possible. A 200 µL drop of the diet solution was placed on the membrane, which was then covered with another layer of stretched Parafilm, enclosing the solution between the 2 layers without air bubbles. Before the experiment, the efficacy of the feeding chamber was validated through a bioassay using the insecticide deltamethrin 2.5%. Adult whiteflies were fed with 100 or 200 ppm of deltamethrin, or water, for 24 h, and mortality rates were recorded. The results confirmed the chamber’s effectiveness, as 100% mortality was observed at both the 100 and 200 ppm concentrations of deltamethrin, and 18% mortality was observed in the control group. Based on these findings, antibiotic treatments were conducted using the same feeding chamber design.

Hundreds of adult whiteflies (0 to 7 d post-emergence) were introduced into each feeding chamber and treated with a 25% sucrose solution (w/v) supplemented with either water or antibiotics. Two types of antibiotic treatments were employed: single antibiotics and antibiotic cocktails. Rifampicin and tetracycline hydrochloride (Hakim Pharmaceutical Co., Tehran, Iran) were used as single antibiotics, while a combination of ampicillin, cefotaxime, and gentamycin (ACG) (Jaber Ebne Hayyan Pharmaceutical Co., Tehran, Iran) constituted the antibiotic cocktail. The selection of antibiotics and their respective applications was informed by evidence derived from prior research studies (Ruan et al. 2006, Ahmed et al. 2010, Xue et al. 2012, Raina et al. 2015, Lv et al. 2018, Wang et al. 2020, Milenovic et al. 2022, Tan et al. 2023). The whiteflies were fed on the single antibiotics and antibiotic cocktail at 50 or 500 μg/ml, respectively. The duration of antibiotic treatments varied: single antibiotics were applied for 2 d, with renewal after 24 h, and the antibiotic cocktail was applied for 4 d, with renewal after 48 h. The selected concentrations and treatment durations were chosen based on findings from previous studies, demonstrating their effectiveness without acting as an antifeedant solution (Ruan et al. 2006, Zhong and Li 2013, Wang et al. 2020, Shan and Liu 2021). Additionally, the selection was guided by the criterion that assay mortality remained below 15% to 25%, as determined through a pretreatment evaluation, which indicated that higher concentrations and extended treatment durations resulted in unacceptably high mortality rates. All antibiotic treatments, as well as subsequent experiments, were conducted in a controlled climate room maintained at 25 ± 2°C, with a photoperiod of 14:10 h (L:D) and 60 ± 5% relative humidity. Following the treatments, the adult whiteflies were collected for subsequent life table and symbiont quantification analyses.

### Life Table Study

Following antibiotic treatment, whitefly pairs, all belonging to the same treatment group, were placed in a cage (20 cm in diameter × 35 cm in length) containing cucumber seedlings. The cage had an 8 cm diameter hole to allow for ventilation. The whiteflies were permitted to oviposit on the cucumber leaves for 24 h. After this egg-laying interval, the adults were removed, and the eggs (42 for ACG, 29 for control, 42 for rifampicin, 45 for tetracycline) were monitored daily. Once the eggs hatched, the first-instar nymphs were initially mobile but soon settled on the undersides of the leaves and remained there until reaching the pupal stage. So, the positions of all nymphs could be marked by a marker for identification. Developmental progress and survival rates were observed daily throughout the entire generation. In the final days of the fourth instar (characterized by red eyes), cucumber leaves were removed from the plants and placed on agar in an insect-rearing box (5 × 1.5 cm). Upon emergence of the adults, they were collected using a small aspirator, and each pair was transferred to a fresh cucumber leaf disc in a separate, isolated insect box. The number of newly laid eggs was recorded and counted until the death of the last adult in the cohort.

### Estimating of Age–Stage, 2-Sex Life Table Parameters

The age–stage, 2-sex life table theory (Chi and Liu 1985, Chi 1988) was utilized to calculate life table parameters using the TWOSEX-MSChart software (version 09/01/2023) and the methods described in the literature (Chi and Liu 1985, Fathipour and Maleknia 2016, Kashkouli et al. 2019, Chi et al. 2023). The following parameters were determined: the age–stage-specific survival rate (*sxj*), which indicates the probability that a newly born individual survives to age *x* and stage *j*; the age-specific survival rate (*lx*), representing the likelihood that an individual survives to age *x*; the age–stage-specific fecundity (*fxj*), which is the average number of eggs produced by females of age *x* and stage *j*; the age-specific fecundity (*mx*), the average number of eggs produced by an individual at age *x*; and the age–stage life expectancy (*exj*), which reflects the expected lifespan of an individual at age *x* and stage *j* (Chi and Liu 1985, Fathipour and Maleknia 2016, Kashkouli et al. 2019, Chi et al. 2023).

Additionally, several population growth parameters were calculated, including the intrinsic rate of increase (*r*), representing the rate at which the population grows; the finite rate of increase (*λ*), indicating how many times the population multiplies per day (with *λ* = e^*r*^); the gross reproductive rate (GRR), which is the total number of eggs produced per individual across a generation, irrespective of individual survival; the net reproductive rate (*R*_0_), reflecting the total number of eggs produced per individual per generation, accounting for survival; and the mean generation time (*T*), which represents the time needed for the population to increase in size by a factor of *R*_0_. To ensure reliable estimates, the standard errors (SE) of these parameters were derived through a bootstrap resampling method with 100,000 iterations. A paired bootstrap test using confidence intervals was conducted (in TWOSEX-MSChart software) to compare the mean estimates of the population parameters obtained from bootstrap resampling.

Furthermore, the longevities of both adult and nymphal stages were calculated, along with the duration of the preoviposition periods. This included the adult preovipositional period (APOP), which is the time from female adult emergence to the first oviposition, and the total preovipositional period (TPOP), the time from egg to first oviposition. The total number of egg masses produced by females throughout their lifetime was also measured as a fecundity indicator. The number of individuals analyzed is presented in Table 1. To assess variations in life stage durations, a one-way analysis of variance (ANOVA) was performed. Additionally, independent sample *t*-tests were conducted to examine differences in preoviposition periods and fecundity across treatments. Multiple comparisons of means were carried out using Tukey’s posthoc test (*P* < 0.05). To account for multiple comparisons, a Bonferroni correction was applied to independent *t*-tests, adjusting the significance threshold (*α*) accordingly. All statistical analyses were performed using SPSS v.16 (SPSS Institute, Chicago, IL). Graphical presentations were generated using GraphPad Prism version 10.

**Table 1.** Mean duration of different stages (± SE) (in days) of the whiteflies, *B. tabaci* and *T. vaporariorum,* in different treatments including a cocktail of ACG, control, tetracycline, and rifampicin. For each stage and whitefly species, different letters indicate statistically significant differences among different treatments (*P* < 0.05, Tukey’s test), ANOVA

Species/Stages	Treatments	Mean duration of different stages (d) ± SE	*N*	*F*-value, df, and *P*-value	Species/Stages	Treatments	Mean duration of different stages (d) ± SE	N	*F*-value, df, and *P*-value
*B. tabaci* Egg	ACG	6.71 ± 0.18ᵃ	42	*F*(3, 154) = 48.17, *P* < 0.0001	*T. vaporariorum* Egg	ACG	7.11 ± 0.08ᵃ	18	*F* (3, 74) = 1.36 *P* = 0.263
	Control	7.76 ± 0.08ᵇ	29			Control	7.05 ± 0.05ᵃ	21	
	Rifampicin	8.24 ± 0.07ᶜ	42			Rifampicin	6.84 ± 0.16ᵃ	19	
	Tetracycline	8.47 ± 0.08ᶜ	45			Tetracycline	6.95 ± 0.09ᵃ	20	
*B. tabaci* N1	ACG	4.17 ± 0.06ᵃ	42	*F* (3, 154) = 346.43 *P* < 0.0001	*T. vaporariorum* N1	ACG	5.33 ± 0.23ᵇ	18	*F* (3, 71) = 3.50 *P* = 0.020
	Control	4.34 ± 0.16ᵃ	29			Control	4.05 ± 0.08ᵃ	21	
	Rifampicin	8.69 ± 0.14ᶜ	42			Rifampicin	4.63 ± 0.43ᵃᵇ	19	
	Tetracycline	5.00 ± 0.10ᵇ	45			Tetracycline	4.68 ± 0.29ᵃᵇ	19	
*B. tabaci* N2	ACG	8.10 ± 0.59ᵇ	42	*F* (3, 147) = 8.67 *P* < 0.0001	*T. vaporariorum* N2	ACG	3.44 ± 0.87ᵃ	9	*F* (3, 54) = 1.51 *P* = 0.222
	Control	4.89 ± 0.50ᵃ	27			Control	2.52 ± 0.13ᵃ	21	
	Rifampicin	6.62 ± 0.62ᵃᵇ	42			Rifampicin	3.25 ± 0.13ᵃ	12	
	Tetracycline	4.80 ± 0.33ᵃ	40			Tetracycline	2.75 ± 0.32ᵃ	16	
*B. tabaci* N3	ACG	7.12 ± 0.52ᵃᵇ	25	*F* (3, 60) = 2.05 *P* = 0.116	*T. vaporariorum* N3	ACG	6.00 ± 0.37ᵇ	6	*F* (3, 43) = 14.99 *P* < 0.0001
	Control	6.12 ± 0.67ᵃᵇ	25			Control	2.90 ± 0.14ᵃ	21	
	Rifampicin	3.33 ± 0.88ᵃ	3			Rifampicin	3.75 ± 0.33ᵃ	12	
	Tetracycline	7.73 ± 1.09ᵇ	11			Tetracycline	5.63 ± 0.87ᵇ	8	
*B. tabaci* Pupa	ACG	10.75 ± 1.33ᵇ	8	*F* (3, 30) = 5.49 *P* = 0.004	*T. vaporariorum* Pupa	ACG	5.50 ± 0.29ᵃ	4	*F* (3, 36) = = 4.86 *P* = 0.006
	Control	6.11 ± 0.73ᵃᵇ	18			Control	6.30 ± 0.24ᵃᵇ	20	
	Rifampicin	1.50 ± 0.50ᵃ	2			Rifampicin	7.60 ± 0.62ᵃᵇ	10	
	Tetracycline	8.17 ± 1.70ᵇ	6			Tetracycline	8.50 ± 0.99ᵇ	6	
*B. tabaci* Preadult	ACG	29.50 ± 0.50ᵃ	2	*F* (2,19) = 4.31 *P* = 0.029	*T. vaporariorum* Preadult	ACG	25.00 ± 0.00ᵃᵇ	2	*F* (3, 25) = 7.74 *P* < 0.0001
	Control	27.64 ± 1.06ᵃ	14			Control	22.93 ± 0.12ᵃ	15	
	Rifampicin	…				Rifampicin	23.38 ± 0.94ᵃ	8	
	Tetracycline	33.17 ± 1.62ᵃ	6			Tetracycline	27.00 ± 0.82ᵇ	4	
*B. tabaci* Adult	ACG	8.50 ± 3.50ᵃ	2	*F* (2,19) = 3.29 *P* = 0.059	*T. vaporariorum* Adult	ACG	3.00 ± 0.00ᵃ	2	*F* (3, 25) = 6.51 *P* = 0.002
	Control	12.86 ± 1.32ᵃ	14			Control	22.73 ± 3.66ᵇ	15	
	Rifampicin	…				Rifampicin	5.00 ± 0.73ᵃ	8	
	Tetracycline	7.83 ± 0.54ᵃ	6			Tetracycline	6.50 ± 1.85ᵃ	4	

### Assessment of Symbiont Abundance Using Quantitative PCR

The relative abundance of symbionts across different treatment groups was evaluated using quantitative PCR (qPCR). The assays were performed on a StepOne Real-Time PCR system (Applied Biosystems, USA) with 20 µl reaction mixtures containing SYBR Green High Rox (Ampliqon, Odense, Denmark), double-distilled water, forward and reverse primers, and DNA extracted from 20 adult females per treatment (or sterile water serving as a negative control). The symbionts *Portiera*, *Arsenophonus*, *Hamiltonella*, and *Rickettsia* were quantified by targeting the amplification of the *16S rRNA*, *23S rRNA*, *16S rRNA*, and *gltA* genes, respectively (see Supplementary Table 2). For normalization, β-actin and NADH genes were used as reference genes for *B. tabaci* and *T. vaporariorum*, respectively (Supplementary Table 2).

Initial optimization of the conditions was performed using gradient PCR, with subsequent analysis of the resulting electrophoresis bands. The qPCR assays were then carried out under the following cycling conditions: an initial activation step at 95 °C for 10 min, followed by 40 cycles consisting of 15 s at 95 °C, 30 s at 60 °C, and 30 s at 72 °C. A melt curve step was also conducted at the end of each run to verify the specificity of the amplification, with fluorescence measurements taken as the temperature gradually increased from 60 to 95 °C.

The optimal DNA concentration for each primer pair was determined by constructing a standard curve for the control, and the slope of the curve was assessed. The cycle threshold (Ct) values were recorded for each primer pair and replicated, with 3 technical replicates per sample. Data were subsequently analyzed and normalized using the ΔΔCt method (Livak and Schmittgen 2001) by comparing the target genes to the reference genes, yielding relative quantities of the symbionts. Statistical comparisons were made between different treatments for each symbiont species. These analyses involved the calculation of means, standard errors, and the assessment of significant differences using ANOVA. Multiple comparisons were conducted using Tukey’s test (*P* < 0.05), with all analyses performed using SPSS software. Graphical representations were generated using GraphPad Prism (Kashkouli et al. 2019).

## Results

### Identification of Whiteflies and Symbionts

The mt-*COI* sequences for *T. vaporariorum* and *B. tabaci* have been deposited in GenBank under accession numbers PP577665 and PP577664, respectively. Additionally, biotype identification of *B. tabaci* was performed using microsatellite markers, revealing that the specimens belonged to biotype B, as confirmed by the PCR fragment size of 200 bp. Molecular analyses further demonstrated that *B. tabaci* harbors a diverse range of symbiotic bacteria, including *Portiera*, *Hamiltonella*, *Arsenophonus*, and *Rickettsia*. In contrast, *T. vaporariorum* was found to be infected exclusively with *Portiera* and *Arsenophonus*.

### Symbiont Titers Following Antibiotic Treatments

**Fig. 1.**
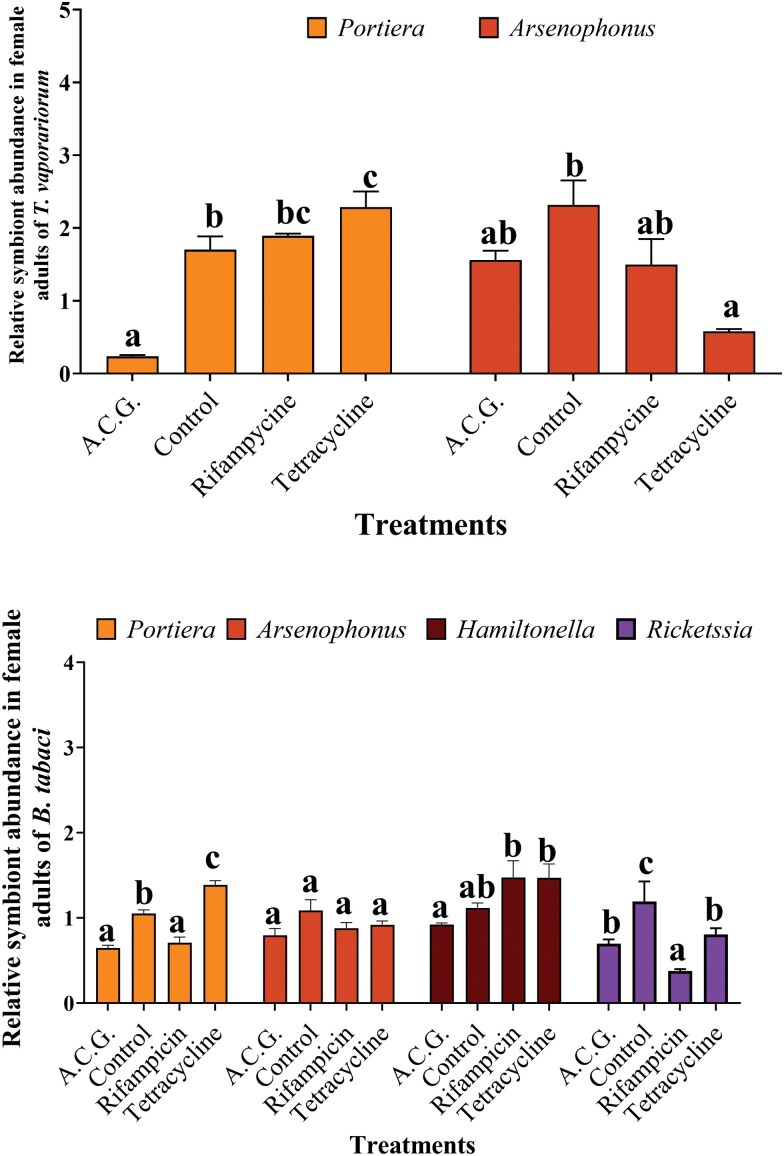
Impact of different treatments including a cocktail of ACG, control, tetracycline, and rifampicin on symbiont levels in *B. tabaci* and *T. vaporariorum* whitefly species. The data are presented as means and standard errors (SE). For each symbiont species, different letters indicate statistically significant differences among different treatments (*P* < 0.05, Tukey’s test), ANOVA.

Analyses of qPCRs revealed significant differences in the symbiont populations of antibiotic-treated whiteflies compared to the control group. Treatments with ACG and tetracycline significantly affected *Portiera* abundance in *B. tabaci* (*F*(3,16) = 49.158, *P* < 0.0001) and *T. vaporariorum* (*F*(3,16) = 39.087, *P* < 0.001). Further comparisons indicated that ACG treatment led to a decrease in *Portiera* abundance, whereas tetracycline treatment resulted in an increase (Fig. 1). Rifampicin treatment also led to a significant reduction in *Portiera* abundance in *B. tabaci* (Fig. 1B).

The symbiont *Arsenophonus* was not significantly affected by any antibiotic treatment, except in tetracycline-treated *T. vaporariorum* (*F*(3,16) = 7.904, *P* = 0.002), where additional analysis revealed a significant reduction in abundance compared to the control (Fig. 1A). The relative density of *Hamiltonella* differed significantly among treatments in *B. tabaci* (*F*(3,16) = 4.306, *P* = 0.021), with post hoc tests indicating a significantly lower abundance in the ACG treatment compared to tetracycline and rifampicin treatments (Fig. 1B). Additionally, all antibiotic treatments significantly affected the abundance of *Rickettsia* in *B. tabaci* (*F*(3,16) = 34.957, *P* < 0.0001), indicating a significant reduction in antibiotic treatments compared with control (Fig. 1B).

### Influence of Antibiotics on the Life Table of Whiteflies

Eggs laid by antibiotic-treated and control whitefly adults were analyzed to assess their fitness, including population growth parameters, stage durations, APOP, TPOP, and fecundity, to construct the life table for both whitefly species. In this study, rifampicin treated *B. tabaci* has lost its ability to being adult, and rifampicin treated *T. vaporariorum* has lost its ability to lay eggs. In addition, treatment with ACG resulted in both whiteflies being unable to lay eggs. Consequently, reproductive parameters such as fecundity rates, fecundity curves, and population growth parameters were not assessed for these treatments.

### Duration of Developmental Stages

The total preadult duration of antibiotic-treated whiteflies was found to be longer compared to the control insects, with significant differences observed only in *T. vaporariorum* treated with tetracycline (Table 1). Stage-specific developmental times also varied across different treatments (Table 1). In *B. tabaci*, significant differences were observed across all stages, except for adult longevity. In *T. vaporariorum*, significant differences were noted in all stages, except for the egg incubation period and second instar duration (Table 1).

### Reproductive Periods and Fecundity

The APOP, TPOP, and fecundity of whiteflies treated with tetracycline were compared to those of the control group. The results showed that neither APOP nor TPOP significantly differed between the treatments and whitefly species (after Bonferroni correction, adjusted *α* = 0.0167). However, fecundity was reduced in tetracycline-treated females of both whitefly species compared to controls (Table 2), although the result did not meet the adjusted significance threshold after correction (Bonferroni *α* = 0.0167).

**Table 2.** Preoviposition periods (d) and fecundity (eggs) of *B. tabaci* and *T. vaporariorum* treated with control or tetracycline. For each parameter and whitefly species, different letters indicate statistically significant differences among different treatments (independent sample *t*-test, Bonferroni-adjusted *α* = 0.0167)

Whitefly species	Parameter	Treatments	Mean (± SE)	*t*	df	*P*
*B. tabaci*	APOP^a^ (day)	Control	4.33 ± 0.803a	0.512	6	0.627
		Tetracycline	3.5 ± 1.5a			
	TPOP^b^ (day)	Control	31.67 ± 1.856a	−1.117	6	0.307
		Tetracycline	36 ± 4a			
	Fecundity	Control	55.5 ± 13.45a	2.031	6	0.088
		Tetracycline	5.5 ± 3.5a			
*T. vaporariorum*	APOP (day)	Control	3 ± 1.84a	−0.822	6	0.443
		Tetracycline	6 ± 3a			
	TPOP (day)	Control	25.83 ± 1.68a	−1.920	6	0.103
		Tetracycline	32 ± 2a			
	Fecundity	Control	105.33 ± 18.45a	2.7	6	0.035
		Tetracycline	13.5 ± 9.5a			

^a^APOP = adult preovipositional period.

^b^TPOP = total preovipositional period (from egg to first oviposition).

### Survival and Fecundity Curves

**Fig. 2.**
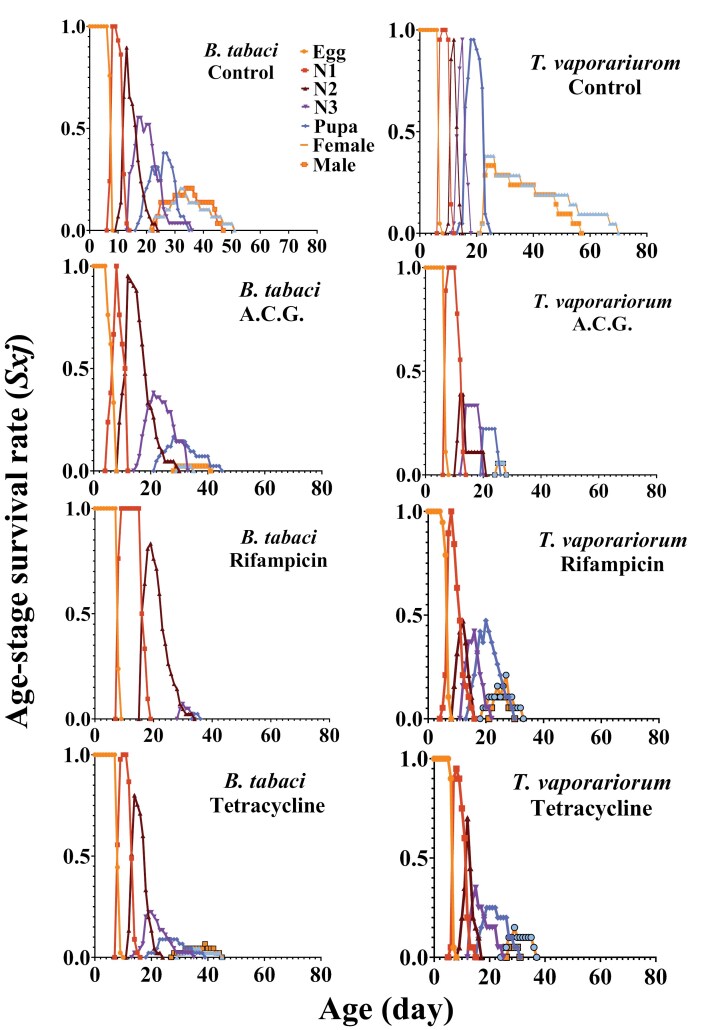
Age–stage-specific survival rate (*sxj*) of the whiteflies, *B. tabaci* and *T. vaporariorum*, in different treatments including a cocktail of ACG, control, tetracycline, and rifampicin.

**Fig. 3.**
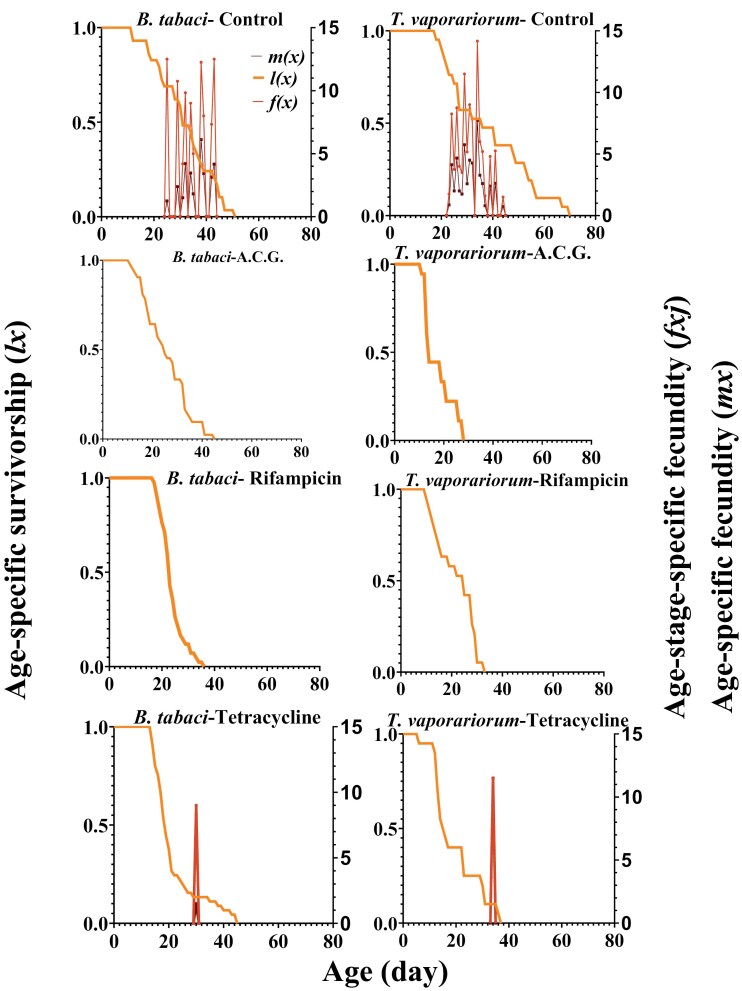
Age-specific survivorship (*lx*), age–stage-specific fecundity (*fxj*), and age-specific fecundity (*mx*) of the whiteflies, *B. tabaci* and *T. vaporariorum,* in different treatments including a cocktail of ACG, control, tetracycline, and rifampicin.

The age–stage-specific survival rates (*sxj*) of whiteflies exhibited different survivorship, distinct trends, and various developmental rates under different treatments (Fig. 2). The age-specific survival rate (*lx*) showed a marked decline in the *lx* curve for antibiotic-treated insects (Fig. 3). Compared to control insects, antibiotic-treated whiteflies exhibited lower survival rates at the same age (Fig. 3).

The age–stage-specific fecundity (*fxj*) and age-specific fecundity (*mx*) of female adults were also compared between control and tetracycline-treated whiteflies. The fecundity (*fx*) curves indicated that oviposition in control insects began on the 25th and 23rd days and lasted for 19 and 22 d for *B. tabaci* and *T. vaporariorum*, respectively (Fig. 3). In contrast, tetracycline-treated whiteflies exhibited single egg-laying patterns starting on the 30th and 34th days for *B. tabaci* and *T. vaporariorum*, respectively (Fig. 3). The peak fecundity (*fxj*) in the control group was 12.5 eggs for *B. tabaci* and 14.17 eggs for *T. vaporariorum*, while in tetracycline-treated whiteflies, the peak fecundity was 9 eggs for *B. tabaci* and 11.5 eggs for *T. vaporariorum* (Fig. 3). Similarly, the *mx* curve reflected the same oviposition durations, with amounts in the tetracycline-treated groups (Fig. 3).

### Life Expectancy Curves

**Fig. 4.**
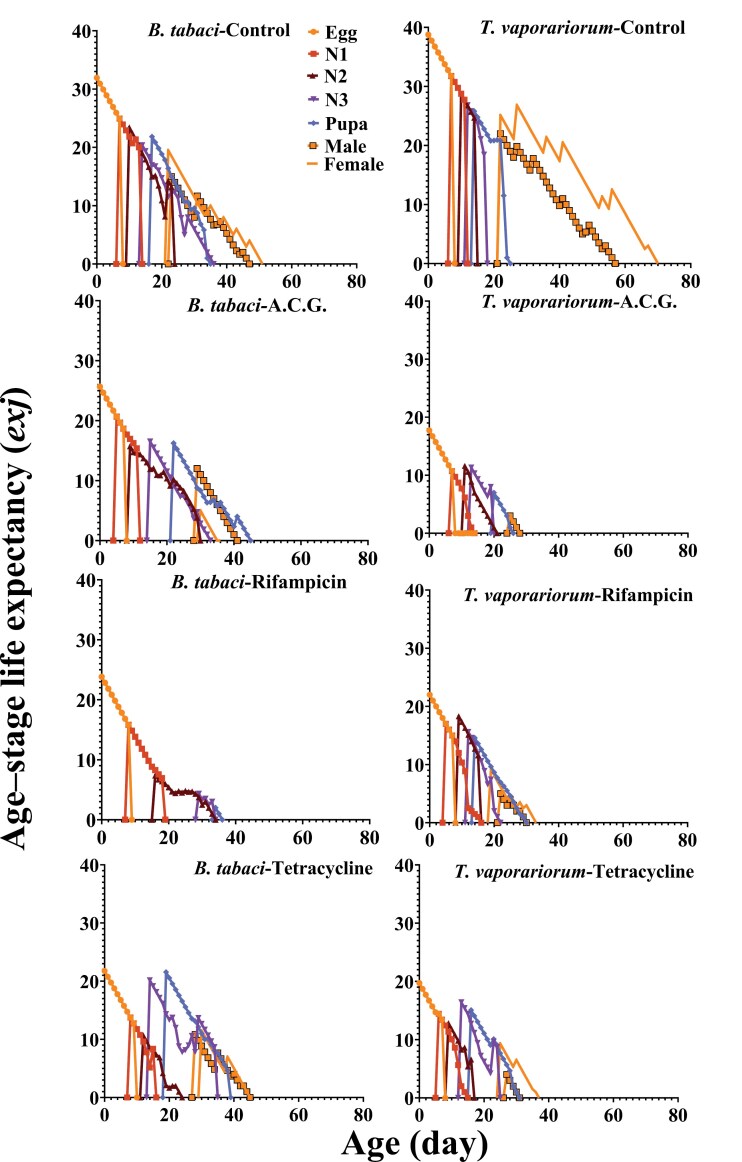
The age–stage life expectancy (*exj*) (day) of the whiteflies, *B. tabaci* and *T. vaporariorum,* in different treatments including: cocktail of ACG, control, tetracycline, and rifampicin.

The age–stage life expectancy (*exj*) of newly hatched eggs for *B. tabaci* under different antibiotic treatments was calculated as follows: ACG (25.76 d), control (31.93 d), rifampicin (23.83 d), and tetracycline (21.76 d) (Fig. 4). For *T. vaporariorum*, the *exj* of newly hatched eggs was 17.22 d for ACG, 38.76 d for the control, 22 d for rifampicin, and 19.7 d for tetracycline (Fig. 4). At the time of female emergence, the *exj* for *B. tabaci* was estimated to be 5 d for ACG, 19.5 d for the control, and 11.5 d for tetracycline treatments. For male emergence in *B. tabaci*, the *exj* was 12 d for ACG, 15 d for the control, and 10.83 d for tetracycline treatments (Fig. 4).

For *T. vaporariorum*, the *exj* at female emergence was 3 d for ACG, 25.12 d for the control, 9.17 d for rifampicin, and 9.33 d for tetracycline treatments. At male emergence, the *exj* was 3 d for ACG, 22 d for the control, 5 d for rifampicin, and 4 d for tetracycline (Fig. 4).

### Mortality Rates

The lowest levels of preadult mortality were observed in the control treatments for both *B. tabaci* and *T. vaporariorum* (Table 3). In contrast, the highest levels of preadult mortality occurred in *B. tabaci* treated with rifampicin and *T. vaporariorum* treated with ACG (Table 3).

**Table 3.** Stage mortality (%) of whiteflies, *B. tabaci* and *T. vaporariorum,* in different treatments including a cocktail of ACG, control, tetracycline, and rifampicin

Whitefly species	Treatments	N1	N2	N3	Pupa	Preadult	Female	Male
*B. tabaci*	ACG	0	40.48	40.48	14.29	95.25	2.38	2.38
	Control	6.9	6.9	24.14	13.79	51.73	20.69	27.59
	Rifampicin	0	92.86	4.76	2.38	100		
	Tetracycline	11.11	64.44	11.11	0	86.66	4.44	8.89
*T. vaporariorum*	ACG	50	16.67	11.11	11.11	88.89	5.56	5.56
	Control	0	0	4.76	23.81	28.57	38.1	33.33
	Rifampicin	36.84	0	10.53	10.53	57.9	26.32	15.79
	Tetracycline	20	40	10	10	80	15	5

### Life Table Parameters

Using the 2-sex life table method, the intrinsic rate of increase (*r*), net reproductive rate (*R*₀), and GRR of tetracycline-treated whiteflies were significantly lower than those of the control group (Table 4). Notably, *r* for *B. tabaci* became negative following tetracycline treatment (Table 4). The finite rate of increase (*λ*) showed significant differences between treatment groups for *B. tabaci*, but no significant differences were observed for *T. vaporariorum* (Table 4). Additionally, there were no significant differences in the mean generation time (*T*) between the treatment groups for either whitefly species (Table 4).

**Table 4.** Population growth parameters (means ± SE) of whiteflies, *B. tabaci* and *T. vaporariorum,* in control or tetracycline treatments. For each parameter and whitefly species, different letters indicate statistically significant differences among treatments (independent sample *t*-test).

Whitefly species	Treatments	*r* (d^−1^)	*λ* (d^−1^)	*R*_0_ (offspring/individual)	*T* (day)	GRR (offspring/individual)
*B. tabaci*	Control	0.07 ± 0.02a	1.07 ± 0.04a	11.48 ± 4.89a	34.48 ± 4.9a	31.5 ± 12.88a
	Tetracycline	−0.04 ± 0.02b	0.96 ± 0.02b	0.24 ± 0.19b	34.661 ± 3.20a	2.17 ± 1.37b
*T. vaporariorum*	Control	0.11 ± 0.02a	1.12 ± 0.04a	30.09 ± 11.46a	30.29 ± 1.49a	52.7 ± 17.97a
	Tetracycline	0.008 ± 0.02b	1.009 ± 0.5a	1.35 ± 1.09b	34.39 ± 16.71a	12.3 ± 8.90b

## Discussion

*B. tabaci* and *T. vaporariorum* were collected and examined for the presence and impact of endosymbionts. Our results demonstrated differences in the composition of symbiotic bacteria among the whitefly species. We identified four main genera of symbiotic bacteria in *B. tabaci*: *Portiera*, *Arsenophonus*, *Hamiltonella*, and *Rickettsia*. However, the symbiotic bacterial composition in *T. vaporariorum* was restricted to *Portiera* and *Arsenophonus*, indicating a lower diversity compared to *B. tabaci*. Both species host *Portiera* as a primary symbiont and *Arsenophonus* as a secondary one*. Portiera* is vital for whiteflies, significantly enhancing their fitness and survival by synthesizing essential amino acids that are lacking in their phloem-based diet (Luan et al. 2015, Tan et al. 2023). Previous research has indicated that *Portiera* enhances the performance of whiteflies and maintains a close nutritional relationship with its host during the acclimatization process to host plants (Hu and Tsai 2020). Additionally, *Arsenophonus* has been identified in *B. tabaci* and *T. vaporariorum*. This symbiont is prevalent among whiteflies and significantly contributes to their biology and ecology, particularly in nutrient provision and reproductive dynamics. The presence of this S-symbiont enhances the whiteflies’ capacity to synthesize essential amino acids, and B-vitamins, enabling them to thrive on nutrient-deficient diets, such as those derived from their primary food source, phloem sap from plants (Upadhyay et al. 2015). Additionally, *Arsenophonus* affects reproductive compatibility among various whitefly species; its presence leads to reproductive incompatibility in interspecies crosses, thereby influencing population dynamics (El Hamss et al. 2021). Conversely, while *Arsenophonus* offers certain benefits, it can also adversely impact whitefly fitness, as evidenced by a decrease in progeny in specific crosses, indicating the complex nature of the relationship between the symbiont and its host (El Hamss et al. 2021).

Several secondary endosymbionts, including *Hamiltonella* and *Rickettsia* have been identified within our *B. tabaci* population. *Hamiltonella* is nearly ubiquitous in *B. tabaci* populations. Research on *B. tabaci* biotypes B and Q indicates that this symbiont is the most frequently observed secondary symbiont, with infection rates of 92.0% in biotype B and 73.3% in biotype Q (Chu et al. 2011). *Hamiltonella* significantly enhances host fitness, particularly in terms of reproductive success; infected whiteflies produce a greater number of eggs and exhibit higher nymphal survival rates (Su et al. 2013). Additionally, infected individuals tend to have accelerated developmental rates, and larger adult body sizes compared to their uninfected counterparts (Su et al. 2013, Shan and Liu 2021). The elimination of *Hamiltonella* from the whitefly host by heat treatment and antibiotics has been shown to influence the sex ratio of the insect population (Shan et al. 2019). *Rickettsia,* another S-symbiont, is predominantly associated with *B. tabaci* and may also play a crucial role in influencing host survival and adaptation (Skaljac et al. 2017, Kareem et al. 2023). *Rickettsia* infection is linked to increased production of juvenile hormones in *B. tabaci*, resulting in enhanced fecundity and a female-biased offspring ratio (Liu et al. 2024). Infected whiteflies exhibit reduced nymphal development times and improved survival rates, thereby contributing to population growth (Himler et al. 2011, Fan et al. 2022). Furthermore, *Rickettsia* enhances the nutritional profile of *B. tabaci*, increasing glycogen and sugar levels, which supports improved growth and reproductive performance (Fan et al. 2022). *Rickettsia* infection also enhances the fitness of the whitefly *B. tabaci* MEAM1 by improving fertility, survival, and development, while also revealing significant changes in gene expression and metabolic pathways linked to energy metabolism and nutrient synthesis (Li et al. 2024). While the advantages conferred by these symbionts are evident, their presence can lead to complex interactions that may differentially impact whitefly populations, highlighting the dynamic nature of these relationships.

Here, we utilized several antibiotics—rifampicin, tetracycline, and a combination of ampicillin, gentamicin, and cefotaxime—to study their effects on the symbionts and host fitness. These antibiotics influence the abundance of symbionts, either increasing or decreasing their populations. Notably, *Portiera* abundance significantly declined following treatment with the A.C.G. in both whiteflies and with rifampicin in *B. tabaci*. These reductions may be involved in our observation that the Rif- and ACG-treated whitefly females could not lay eggs. This finding aligns with previous studies indicating that rifampicin reduces the population of *Portiera* (Zhang et al. 2015, Shan et al. 2016, Milenovic et al. 2022, Tan et al. 2023). Conversely, we observed a significant increase in *Portiera* abundance after tetracycline treatment in both whitefly species. This phenomenon can be explained by the dynamic interactions between P-symbionts and S-symbionts, as they co-inhabit the bacteriocytes and compete for cellular space and host-derived resources, a process observed in whiteflies (Skaljac et al. 2010, Tan et al. 2023). Thus, the reduction of S-symbiont populations by tetracycline treatment likely provided additional resources for *Portiera* as a P-symbiont.

Moreover, other bacterial symbionts, such as *Arsenophonus* and *Rickettsia*, exhibited significant declines in abundance following tetracycline treatment in *T. vaporariorum* and antibiotic treatment in *B. tabaci*, respectively. Some studies confirmed that tetracycline reduce the abundance of symbiotic bacteria and decrease the size of mycetomes (Costa et al. 1993, Ruan et al. 2006, Ahmed et al. 2010). In addition, tetracycline effectively targets *Arsenophonus* leading to potential residual effects on the host’s reproductive capabilities, overall fitness (Zhong and Li 2013), and transmission efficiency of Cotton Leaf Curl Virus (CLCuV) by whiteflies (Kaur et al. 2022). The effects of antibiotics on the *Rickettsia* symbiont in whiteflies, particularly through rifampicin treatment, demonstrate significant impacts on symbiont abundance. Specifically, rifampicin treatment leads to a marked reduction in *Rickettsia* density—although it does not completely eradicate it—especially in the offspring of treated adults, with significant reductions observed in the F1 generation (Zhang et al. 2015, Shan et al. 2016).

Fluctuations of primary and secondary symbiont titers in whiteflies following an antibiotic treatment negatively affect the host’s fitness. Here we utilized the age–stage 2-sex life table to understand the impact of antibiotics on the survival, development, and reproduction rates of insect populations (Chi et al. 2020, 2023). Our result indicated that antibiotic treatments affected preadult durations, fecundity, life expectancy, and some biological parameters including APOP, TPOP, *r*, *R*_0_ and GRR. In our present result, the preadult durations of whiteflies in the progeny of parents exposed to antibiotics were longer than progeny from control parents. Similar results were observed in the preadult duration of F1 *Aleurocanthus camelliae,* which was significantly longer in rifampicin treated insects (Tan et al. 2023), and development of *B. tabaci* juveniles, which was significantly longer in tetracycline-treated insects (Raina et al. 2015). In addition, in some other hemipteran bugs such as *Graphosoma lineatum* L. (Karamipour et al. 2021), *Brachynema germari* Kolenati, and *Acrosternum heegeri* Fieber (Kashkouli et al. 2019), the preadult duration tended to be prolonged in symbiont-treated insects. After antibiotic treatment, the preoviposition periods (TPOP and APOP) were not significantly affected. The same result was observed in *B. tabaci* Q populations, infected and uninfected with *Cardinium* (Fang et al. 2014). In *A. camelliae*, the TPOP of the aposymbiotic insect was extended compared with the control, while the APOP had no significant difference between the treatments (Tan et al. 2023). In addition, the whiteflies experienced reduced fecundity in response to tetracycline treatment. A similar result was observed in *A. camelliae*, in which the symbiont-manipulated group had decreased fecundity rate rather than the control group (Tan et al. 2023). Poor reproductive success was also reported in antibiotic-treated *B. tabaci*, with F1 adults laying few eggs, all of which failed to hatch (Zhang et al. 2015, Shan et al. 2016). But in some other studies, symbiont infections had no effect (Fang et al. 2014) or negative effect (Raina et al. 2015) on *B. tabaci* fecundities. In general, the life expectancy at the time of egg hatching and adult emergence was the shorter in the antibiotic-treated whiteflies in comparison to the control, agreed with shorter life expectancy of *G. lineatum* aposymbiont (Karamipour et al. 2021). In addition, antibiotic treatments led to higher preadult mortality of both whiteflies rather than the control, which the highest levels of preadult mortality occurred in *B. tabaci* treated with rifampicin and *T. vaporariorum* treated with ACG. These findings align with the observed reduction in *Portiera* levels in these antibiotic-treated whiteflies, suggesting that the increased mortality may be attributed to the disruption of *Portiera* symbionts.

Here, we observed a larger slope of the *lx* curve in antibiotic-treated groups than control, mainly due to the lower egg hatchability and higher mortality of the nymph stages agreed with what was observed in *A. camelliae* (Tan et al. 2023). Concerning the *fx* and *mx* curves, the control group of whiteflies had an earlier age of starting oviposition and higher fecundity than tetracycline-treated whiteflies. The tetracycline treatment clearly shortened the oviposition period and reduced fecundity. We also found a significant decrease in other biological parameters such as *r*, *R*_0_, and GRR in the tetracycline-treated whiteflies compared with the control. In addition, we observed a significant decrease in *λ* parameter of *B. tabaci* in response to tetracycline treatment. These changed biological traits negatively affected the development of whiteflies, ultimately resulting in the reduction of the offspring population. In *A. camelliae*, *r*, *R*_0_, and *λ* were significantly lower in the aposymbiotic group than in the control population (Tan et al. 2023). In *Aphis gossypii*, reductions of the secondary symbionts (*H. defensa* and *Arsenophonus*) negatively affected *r*, *R*_0_, *λ*, *T*, and *DT* (Ayoubi et al. 2020). In *G. lineatum, r*, *R*_0_, and GRR were significantly lower in the aposymbiotic rather than the control (Karamipour et al. 2021). In *B. germari* and *A. heegeri*, aposymbiotic cohorts had significantly lower *r*, *R*_0_, and *GRR* values in comparison with control cohorts (Kashkouli et al. 2019). In contrast, *r*, *R*_0_, and *λ* of the *Cardinium*-uninfected *B. tabaci* population were significantly higher than that of the *Cardinium*- infected *B. tabaci* population (Fang et al. 2014). Co-infection with *Hamiltonella* and *Cardinium* can lead to reduced fecundity and a male-biased sex ratio, which negatively impacts the intrinsic rate of increase in *B. tabaci* (Zhao et al. 2018).

Taken together, this study underscores the critical role of endosymbionts in shaping the fitness and population dynamics of *B. tabaci* and *T. vaporariorum*. The observed differences in symbiont composition between these 2 whitefly species suggest that *B. tabaci* benefits from a broader diversity of endosymbionts, which likely contributes to its enhanced ecological success. Antibiotic treatments revealed significant fitness costs for the whiteflies, including reductions in reproductive success, developmental rates, and overall survival, however, the side effects of antibiotics should not be ignored. Regarding a practical perspective, the reduction of symbionts and the resulting fitness deficits in whitefly hosts indicate that antibiotic-based treatments could serve as a rational alternative to conventional insecticides, potentially integrated into IPM strategies. However, the long-term effects of such treatments, particularly across multiple generations, remain poorly understood and warrant further investigation to evaluate the risk of antibiotic resistance and the impact on beneficial microbial communities. Future research should focus on elucidating the specific physiological roles of different symbionts and the mechanisms through which they influence host fitness. These insights will be essential for advancing IPM approaches that can sustainably mitigate the threat posed by whiteflies in agricultural contexts.

## Supplementary Material

toaf136_Supplementary_Tables_1-2
