# Supplementary material for: Effects of antibiotic treatments on symbiotic bacteria and life history traits of Bemisia tabaci and Trialeurodes vaporariorum (Hemiptera: Aleyrodidae): implications for pest control strategies
Source: J Econ Entomol. 2025 Sep 11;118(6):3190–201. doi: 10.1093/jee/toaf136 (PMC12698220; doi:10.1093/jee/toaf136)
Supplement: toaf136_Supplementary_Tables_1-2 [file toaf136_supplementary_tables_1-2.docx]

**Effects of Antibiotic Treatments on Symbiotic Bacteria and Life History Traits of *Bemisia* *tabaci* and *Trialeurodes vaporariorum* (Hemiptera: Aleyrodidae): Implications for Pest Control Strategies
Marzieh Kashkouli^1^ , Jahangir Khajehali^2^**

1- Research Institute for Biotechnology and Bioengineering, Isfahan University of Technology, Isfahan 84156‑83111, Iran

2-Department of Plant Protection, College of Agriculture, Isfahan University of Technology, Isfahan 84156‑83111, Iran

Corresponding Author: Marzieh Kashkouli, [mkashkouli@iut.ac.ir](mailto:mkashkouli@iut.ac.ir), +9833912763

| **Supplementary Table 1**. Oligonucleotide primers used for symbiont identification in PCR | | | | |
| --- | --- | --- | --- | --- |
| Targeted gene | Primers | Amp. Size (bp) | Sequences (5' - 3') | References |
| *T. vaporariorum* and *B. tabaci mtCO1* | C1-J-2195 | 850 | TTGATTTTTTGGTCATCCAGAAGT | (Frohlich et al., 1999; Liu et al., 2020) |
|  | L2-N-3014 |  | TCCAATGCACTAATCTGCCATATTA |  |
| *B. tabaci* B/Q biotypes micro-sattelite | Bem23-F | B biotype: 200 bp  Q biotype: 400 bp | CGGAGCTTGCGCCTTAGTC | (De Barro et al., 2003; Skaljac et al., 2010) |
|  | Bem23-R |  | CGGCTTTATCATAGCTCTCGT |  |
| *Portiera 16S rRNA* | 28F | 1000-1100 | TGCAAGTCGAGCGGCATCAT | (Gao et al., 2014) |
|  | 1098R |  | AAAGTTCCCGCCTTATGCGT |  |
| *Arsenophonus 23S rRNA* | Ars23S.1 | 600 | CGTTTGATGAATTCATAGTCAAA | (Cass et al., 2014; Kapantaidaki et al., 2015) |
|  | Ars23S.2 |  | GGTCCTCCAGTTAGTGTTACCCAAC |  |
| *Wolbachia 16S rRNA* | Wol16S-f | 600 | CGGGGGAAAAATTTATTGCT | (Kapantaidaki et al., 2015) |
|  | Wol16S-r |  | AGCTGTAATACAGAAAGTAAA |  |
| *Hamiltonella 16S rRNA* | Ham-F | 1000 | TGAGTAAAGTCTGGGAATCTGG | (Gao et al., 2014; Liu et al., 2020) |
|  | Ham-R |  | CCCGGGAACGTATTCACCGTAG |  |
| *Rickettsia 16S rRNA* | RB_F | 900 | GCTCAGAACGAACGCTATC | (Kapantaidaki et al., 2015) |
|  | RB_R |  | GAAGGAAAGCATCTCTGC |  |
| *Cardinium 16S rRNA* | CFB_F | 400 | GCGGTGTAAAATGAGCGTG |  |
|  | CFB_R |  | ACCTMTTCTTAACTCAAGCCT |  |
| *Fritschea 23S rRNA* | U23F | 600 | GATGCCTTGGCATTGATAGGCGATGAAGGA |  |
|  | 23SIGR |  | TGGCTCATCATGCAAAAGGCA |  |

| **Supplementary Table 2**. Oligonucleotide primers used for symbiont quantification in qPCR | | | | |
| --- | --- | --- | --- | --- |
| *Hamiltonella16S rRNA* | qHam-F | 243 | GCATCGAGTGAGCACAGTTT | (Pan et al., 2013) |
|  | qHam-R |  | TATCCTCTCAGACCCGCTAGA |  |
| *Rickettsia gltA* | qRick-F | 200 | TGGTATTGCATCGCTTTGGG | (Wang et al., 2020) |
|  | qRick-R |  | TTTCTTTAAGCACTGCAGCACG |  |
| *Arsenophonus*  *23S rRNA* | qArs-F | 174 | ATGGTGCCGTAACTTCAGGA |  |
|  | qArs-R |  | TAACCTTACAGCACCTGGCA |  |
| *Portiera* symbiont of *Trialeurodes vaporariorum 16S rRNA* | Tport-F | 168 | GTAATACGGAGGGTGCAAGC |  |
|  | Tport-R |  | ATTTCACCGCTACACCTGGA |  |
| *Trialeurodes vaporariorum* *NADH* | Tnadh-F | 99 | GCGCTTTGCTTTACCTTTGT |  |
|  | Tnadh-R |  | GGTGGTCGGCAAGATTTGTT |  |
| *Portiera* symbiont of *Bemisia tabaci* *16S rRNA* | Bport-F | 200 | GTGGGGAATAACGTACGG | (Bing et al., 2013; Tan et al., 2023) |
|  | Bport-R |  | CTCAGTCCCAGTGTGGCTG |  |
| *Bemisia tabaci* *Actin* | Bactin-F | 130 | TCTTCCAGCCATCCTTCTTG | (Liu et al., 2020) |
|  | Bactin-R |  | CGGTGATTTCCTTCTGCATT |  |

References

Bing XL, Yang J, Fein JEZ, et al. 2013. Characterization of a newly discovered symbiont of the whitefly Bemisia tabaci (Hemiptera: Aleyrodidae). Appl. Environ. Microbiol. 79:569–575. <https://doi.org/10.1128/AEM.03030-12>.

Cass BN, Mozes-Daube N, Iasur-Kruh L, et al. 2014. Bacterial endosymbionts in field-collected samples of Trialeurodes sp. nr. abutiloneus (Hemiptera: Aleyrodidae). Res. Microbiol. 165:77–81. <https://doi.org/10.1016/j.resmic.2014.01.005>.

Frohlich DR, Torres-Jerez I, Bedford ID, et al. 1999. A phylogeographical analysis of the Bemisia tabaci species complex based on mitochondrial DNA markers. Mol. Ecol. 8:1683–1691. <https://doi.org/10.1046/j.1365-294x.1999.00754.x>.

Gao RR, Zhang WP, Wu HT, et al. 2014. Population structure of the greenhouse whitefly, Trialeurodes vaporariorum (Westwood), an invasive species from the americas, 60 years after invading China. Int. J. Mol. Sci. 15:13388–13400. <https://doi.org/10.3390/ijms150813388>.

Kapantaidaki DE, Ovčarenko I, Fytrou N, et al. 2015. Low levels of mitochondrial DNA and symbiont diversity in the worldwide agricultural pest, the greenhouse whitefly Trialeurodes vaporariorum (Hemiptera: Aleyrodidae). J. Hered. 106:80–92. <https://doi.org/10.1093/jhered/esu061>.

Pan H, Chu D, Liu B, et al. 2013. Relative amount of symbionts in insect hosts changes with host-plant adaptation and insecticide resistance. Environ. Entomol. 42:74–78.
